# Costs and Effects of Abdominal versus Laparoscopic Hysterectomy: Systematic Review of Controlled Trials

**DOI:** 10.1371/journal.pone.0007340

**Published:** 2009-10-05

**Authors:** Claudia B. M. Bijen, Karin M. Vermeulen, Marian J. E. Mourits, Geertruida H. de Bock

**Affiliations:** 1 Department of Gynecologic Oncology, University Medical Center Groningen, University of Groningen, Groningen, The Netherlands; 2 Department of Epidemiology, University Medical Center Groningen, University of Groningen, Groningen, The Netherlands; Assiut University Hospital, Egypt

## Abstract

**Objective:**

Comparative evaluation of costs and effects of laparoscopic hysterectomy (LH) and abdominal hysterectomy (AH).

**Data sources:**

Controlled trials from Cochrane Central register of controlled trials, Medline, Embase and prospective trial registers.

**Selection of studies:**

Twelve (randomized) controlled studies including the search terms costs, laparoscopy, laparotomy and hysterectomy were identified.

**Methods:**

The type of cost analysis, perspective of cost analyses and separate cost components were assessed. The direct and indirect costs were extracted from the original studies. For the cost estimation, hospital stay and procedure costs were selected as most important cost drivers. As main outcome the major complication rate was taken.

**Findings:**

Analysis was performed on 2226 patients, of which 1013 (45.5%) in the LH group and 1213 (54.5%) in the AH group. Five studies scored ≥10 points (out of 19) for methodological quality. The reported total direct costs in the LH group ($63,997) were 6.1% higher than the AH group ($60,114). The reported total indirect costs of the LH group ($1,609) were half of the total indirect in the AH group ($3,139). The estimated mean major complication rate in the LH group (14.3%) was lower than in the AH group (15.9%). The estimated total costs in the LH group were $3,884 versus $3,312 in the AH group. The incremental costs for reducing one patient with major complication(s) in the LH group compared to the AH group was $35,750.

**Conclusions:**

The shorter hospital stay in the LH group compensates for the increased procedure costs, with less morbidity. LH points in the direction of cost effectiveness, however further research is warranted with a broader costs perspective including long term effects as societal benefit, quality of life and survival.

## Introduction

Traditionally abdominal hysterectomy (AH) is the standard procedure for gynecological malignancy and several benign indications and remains the ‘fallback option’ if the uterus cannot be removed by another approach. While the abdominal surgical approach is an accepted effective treatment, it is associated with substantial morbidity, mostly wound problems on the short-term ([1]–[3] and incisional hernias on the long-term [4]. A good alternative approach for patients with an indication for removal of the uterus is by laparoscopy. The laparoscopic approach to hysterectomy has evolved over the last 20 years. In 1984, Kurt Semm [5] introduced laparoscopic assistance to complicated vaginal hysterectomy and this was followed by laparoscopically assisted vaginal hysterectomy as described by Harry Reich [6]. Three types of laparoscopic hysterectomy (LH) are currently practiced: Laparoscopically Assisted Vaginal Hysterectomy (LAVH), Total Laparoscopic Hysterectomy (TLH) and Laparoscopically Assisted Supracervical Hysterectomy (LASH). The laparoscopic approach has the advantages of laparotomy, i.e. possibility of thorough abdominal inspection to assess the abdominal cavity for extra-uterine spread and collection of peritoneal fluid for cytology. Moreover, since patients do not have a large abdominal wound, the laparoscopic approach results in a shorter hospital stay, less abdominal wound morbidity and quicker return to activity in daily life [1], [2]. Nevertheless, for several reasons laparoscopy is not an established procedure for all indications for abdominal hysterectomy yet. The first reason is inexperience of surgeons with this advanced laparoscopic procedure, which results in a higher peri-operative complication rate during the learning curve [7]. Next reason is economic: higher per-operative costs, longer operation time, expensive surgical (disposable) equipment and extra costs in converted procedures. However, laparoscopic approaches to hysterectomy offer the prospect of improved outcomes and gains in cost effectiveness through better and quicker convalescence and shorter length of inpatient stay.

A combination of the development of expensive new treatments for patients, the limited budgets available for health care and the increasing demands on the health system has led to a need to evaluate the costs in addition to clinical effects, in order to make rational decisions regarding the acceptance of new treatments into the health service.

Therefore, it is important to determine the actual costs of a new treatment and to compare those with the costs of the standard treatment, in order to give this new method a chance to be fairly judged and further propagated. With the exception of some observational studies [8]–[10] and small randomized trials [11], [12], little is known about the costs and cost effectiveness of LH relative to the conventional abdominal approach. Our aim of this study is to pool the data of controlled trials and review whether this new health technology, laparoscopic hysterectomy, provides ‘good value for money’ in comparison to the conventional procedure.

## Methods

### Search strategy

**Figure 1:**
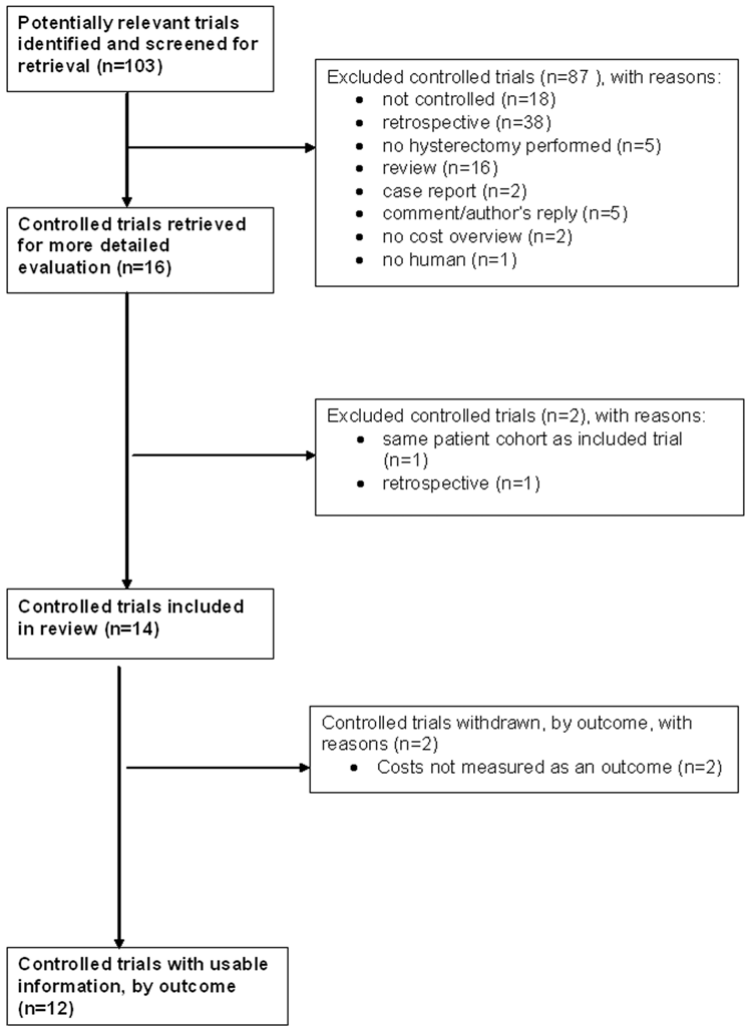
Flowchart of included studies.

The methodology of the review was according to the QUORUM statement [13] (appendix S1). Studies comparing the costs and cost effectiveness of LH versus AH were sought from a systematic review of the literature. The electronic databases Medline, Embase and the Cochrane library database were searched for relevant articles between the years 1990 and 2008. Prospective clinical trial registers were also searched for the same keywords. Search terms used were: costs, laparoscopy, laparotomy and hysterectomy. The references of all relevant articles were hand-searched for any previously missed articles. The results of the search in electronic databases and the hand search were indicated in the flowchart (figure 1).

### Selection of trials

All studies that compared costs between AH and all forms of LH were included in this review. Studies were excluded from the review if they made comparisons other than those specified above. There was no restriction to any language. Furthermore, reviews, letters, author's replies, retrospective studies and case series with no control group were excluded, whereas prospective controlled studies and randomized controlled trials were included. Papers were independently appraised by two authors (KV, CB) in terms of their methodology and design by using a predetermined protocol and graded as to the level of evidence (table 1 and 2). Differences were resolved by discussion between the authors.

**Table 1:** Methodological quality assessment*.

Category	Subcategory	score
***Generation of allocation sequence***	Computer generated random number	2
	Not described	1
***Allocation concealment***	Central randomization	3
	Sealed enveloped or similar	2
	Not described or inadequate	1
***Outcome assessor blindness***	Blinded for treatment arm	2
	Inadequate blinding	1
	Not described or no double-blinding	0
***Description of withdrawals and dropouts***	Numbers and reasons are described	2
	Number or reasons are not described	1
	Numbers and reasons are not described	0
***Efficacy of randomization/controlling***	Pretreatment variables in tabular form	2
	Balance of pretreatment variables mentioned but not in tabular form	1
	No information report	0
***Analysis include an intention-to treat analysis***	Intention-to treat analysis described	2
	Unclear	1
	No intention-to-treat analysis used	0
***All important and relevant costs for each arm***	For direct and indirect costs	2
	For direct or indirect costs	1
	Incomplete cost analysis	0
***Valid measurements and detailed description of costs***	Yes	2
	Unclear	1
	No	0
***Relevant sensitivity analysis***	Sensitivity analysis described and in tabular/graphical form	2
	Sensitivity analysis described, no tabular/graphical form	1
	No sensitivity analysis described	0

* = analogously of Jadad score

**Table 2:** Study characteristics.

Author	Design	Cost analysis	Cost Perspective	Participants (n = )	Indication	Interventions	Quality assessment score
***Abdelmonem 2006***	CCT; Multi centre	Cost consequence	Hospital	177	Benign	AH	5
						LH [LAVH, LSH, TLH]	
						VH	
***Van den Eeden 1998***	CCT; Multi centre	Cost consequence	Hospital	287	Benign/Malignant	AH	9
						LH [LAVH]	
						VH	
***Ellstrom 1998***	RCT; Single centre	Cost consequence	Societal	143	Benign	AH +/− BSO	13
						LH [TLH] +/− BSO	
***Eltabbakh 2001***	CCT; Single centre	Cost consequence	Hospital	147	Malignant	AH +BSO +/− LN	10
						LH [LAVH] +BSO +/− LN	
***Falcone 1999***	RCT; Single centre	Cost minimization	Hospital	48	Benign	AH	11
						LH [LAVH]	
***Howard 1993***	CCT; Single centre	Cost consequence	Hospital	30	Benign	AH	2
						LH [LAVH]	
***Kung 1996***	CCT; Single centre	Cost	Hospital/Insurance	301	Benign	AH	6
						LH [LAVH]	
***Leng 2004***	CCT; Single centre	Cost	Societal	54	Benign	AH	5
						VH	
						LH	
***Lumsden 2000***	RCT; Multi centre	Cost consequence	Hospital	190	Benign	AH	17
						LH [LAVH]	
***Raju 1994***	RCT; Single centre	Cost minimization	Hospital	80	Benign	AH +BSO	7
						LH [LAVH] +BSO	
***Sculpher 2004***	RCT; Multi centre	Cost utility	Health care provider	1380	Benign	VH	15
						AH	
						LH [LAVH, LSH, TLH]	
***Summit 1998***	RCT; Multi centre	Cost	Hospital	65	Benign	AH	11
						LH [LAVH]	

*CCT =  controlled clinical trial; RCT =  randomized controlled trial; AH =  abdominal hysterectomy; LH =  laparoscopic hysterectomy; LAVH = laparoscopic assisted vaginal hysterectomy; LSH =  laparoscopic supracervical hysterectomy; TLH = total laparoscopic hysterectomy; VH = vaginal hysterectomy; BSO =  bilateral salpingo-oophorectomy; LN =  lymph nodes.*

The search strategy identified 16 studies [11],[12],[14]–[27] that compared the costs of laparoscopic hysterectomy and abdominal hysterectomy. Four [23]–[25], [27] of these were not suitable for further reviewing according to our in- and exclusion criteria. The flow of studies included in the analysis is presented in figure 1 (based on the QUORUM flowchart) [13].

### Data management

All data were extracted independently by two reviewers (KV, CB) and differences of opinion were resolved by consensus. Extracted data are presented in detail in appendix S2 and general study characteristics are shown in table 2. The economic impact of a surgical procedure can be divided into direct medical costs and indirect costs. Direct costs relate to health care costs and include those relating to the hospital stay, operative procedure and treatment-related complications. Procedure costs relate to the instruments and equipment used for the procedure, the duration (including anesthetic time), staff costs and overheads costs of the theatre. Indirect costs are the societal (because of a loss of productivity) and individual costs relating to a patient's absence from work or normal activities; this also includes costs of carers. Wherever possible, costs have been classified in this manner, although some of the studies vary in their definitions.

### Analysis of (inter study) costs and effects

#### Reported costs

The type of cost analysis, perspective of cost analyses and all separate cost components of the studies were assessed (table 2 and 3). In case a vaginal hysterectomy arm was included, these data were excluded from further analysis. The direct and indirect costs as reported by the authors between the procedures and within studies were specified and expressed in US dollar (table 3). No formal meta-analysis could be performed because of the heterogeneity in the selected studies in terms of date of publication, currency in which costs were calculated, statistical method used, disease process examined and particularly the range of cost components that were included and primary effect measures which were taken into account.

**Table 3:** Actual direct and/or indirect costs per treatment arm.

Author	Currency	LH		AH		Cost components
		*direct*	*Indirect*	*direct*	*indirect*	
***Abdelmonem 2006***	US dollar	$16,451↑	n.i.	$15,145	n.i.	Hospital cost, anesthesia
***V. d. Eeden 1998***	US dollar	$8,099 ↓	n.i.	$9,135	n.i.	Preoperative costs, hospital costs (procedure, hospital stay, physician fee), postoperative costs.
***Ellstrom 1998***	SEK	$3,169 ↑	$1,411↓	$3,116	$2,838	Surgery, services from other departments, hospital stay, postoperative visits, sick leave
***Eltabbakh 2001***	US dollar	$13,051↑	n.i.	$11, 028	n.i.	Surgeon's fee, anesthesiologist's fee, operating room cost, hospital cost
***Falcone 1999***	US dollar	$+277 ↑ *	n.i.	$0 *	n.i.	Operating room cost, anesthesia, postoperative cost
***Howard 1993***	US dollar	$3,926 ↓	n.i.	$4,524	n.i.	Operating equipment, operating time, anesthesia, non operating costs
***Kung 1996***	NT dollar	$1,819 ↑	n.i.	$1,566	n.i.	Laboratory test, examination charges, surgery and material fees, anesthesia, nursing care, ward fee
***Leng 2004***	Yuan	$876 ↑	$198 ↓	$749	$301	Operating costs, outpatient department visits, readmissions, hospital stay (incl. medication), sick leave, transport costs
***Lumsden 2000***	UK pound	$3,462 ↑	n.i.	$2,733	n.i.	Operation costs, hospital stay, readmissions
***Raju 1994***	UK pound	$1,909 ↓	n.i.	$2,652	n.i.	Disposables, hospital stay, operating time
***Sculpher 2004***	UK pound	$2,797 ↑	n.i.	$2,492	n.i.	Ward fee, theatre (staff, overhead, disposables), visits (GP, outpatient department)
***Summit 1998***	US dollar	$8,161 ↑	n.i.	$6,974	n.i.	Not specified
**Subtotal RCT**		$**19,775**↑	$**1,411**↓	$**17,967**	$**2,838**	
**Total**		$**63,997** ↑	$**1,609** ↓	$**60,114**	$**3,139**	

n.i. =  not included; * =  difference in means; Exchange rates of year of performing study ( http://www.x-rates.com/cgi-bin/hlookup.cgi *).*

#### Estimated costs and effects

To be able to draw a global conclusion with regard to costs and the ratio between costs and effects, overall treatment costs were estimated based on costs in the current situation in Dutch hospitals (table 4 and 5). Costs of the procedure were estimated based on personnel costs ($4.14 or €3.12/min), overhead and housing (45%) according to the Dutch guidelines for costs studies. Costs of disposables ($1,328 or €1,000: only LH) were globally assessed based on the current Dutch protocol [28]. Costs of hospital stay were based on Dutch standard prices (mean of general hospital and university medical center: $571/€430). The price level used was that of 2007 and costs are expressed in US dollars ($). Since information on other cost categories was scarce in most studies, those costs were not included in the present estimation. As primary effect measure the overall complication rate as well as the major complication rate was taken. The division between and definition of major and minor complications were subtracted from the original article. The complication rate consists of the reported complications added up with the amount of re-interventions performed. Conversions to laparotomy were not considered as complications.

**Table 4:** Costs and effects of laparoscopic hysterectomy.

Author	Costs		Effects		
	*Procedure (time in minutes)*	*Hospital stay (days)*	Complication rate * (Major)	Complication rate * (Minor)	Complication rate * (Overall)
***Abdelmonem 2006***	157.8	1.5	9.8 (5/51)	7.8 (4/51)^a^	13.7 (7/51)
***Van den Eeden 1998***	200.6	1.8	0.0 (0/56)	0.0 (0/56)	0.0 (0/56)‡
***Ellstrom 1998***	148.0	2.5	26.8 (19/71)**	n.s.	26.8 (19/71)
***Eltabbakh 2001***	190.5	2.5	11.6 (10/86)	n.s.	11.6 (10/86)
***Falcone 1999***	180.0	1.5	39.1 (9/23)	n.s.	39.1 (9/23)
***Howard 1993***	169.0	3.7	13.3 (2/15)	n.s.	13.3 (2/15)
***Kung 1996***	134.0	4.9	15.2 (21/138)	n.s.	15.2 (21/138)
***Leng 2004***	n.a.	5.1	n.a.	n.a.	n.a.
***Lumsden 2000***	81.9	4.0	5.3 (5/95)	n.s.	8.0
***Raju 1994***	100.0	3.5	5.0 (2/40)	n.s.	5.0 (2/40)
***Sculpher 2004***	108.1	4.0	10.1 (59/584)**	25.2 (147/584)**	n.s.
***Summit 1998***	179.8	2.1	20.6 (7/34)	n.s.	20.6 (7/34)‡
**Weighted mean RCT**	**113.0**	**3.7**	**17.8**	**25.2**	**19.9**
**Weighted mean**	**127.7**	**3.6**	**14.3**	**11.0**	**15.3**

n.s.  =  not specified; n.a.  =  not available; * =  percentage of patients with complications; ** =  data derived from original article; ^a^  =  converted patients reported on complication level instead of patient level. Therefore these patients can not be excluded; ‡ =  number of complications reported.

**Table 5:** Costs and effects of abdominal hysterectomy.

Author	Costs		Effects		
	*Procedure (time in minutes)*	*Hospital stay (days)*	Complication rate * (Major)	Complication rate * (Minor)	Complication rate * (Overall)
***Abdelmonem 2006***	127.2	3.7	10.0 (5/50)	28.0 (14/50)	32.0 (16/50)
***Van den Eeden 1998***	151.4	3.3	1.2 (2/164)	14.0 (23/164)	15.2 (25/164)‡
***Ellstrom 1998***	93.1	5.0	33.3 (24/72)**	n.s.	33.3 (24/72)**
***Eltabbakh 2001***	132.8	5.2	8.8 (5/57)	n.s.	8.8 (5/57)
***Falcone 1999***	130.0	2.5	23.8 (5/21)	n.s.	23.8 (5/21)
***Howard 1993***	119.0	5.2	40.0 (6/15)	n.s.	40.0 (6/15)
***Kung 1996***	112.0	5.2	17.8 (28/157)	n.s.	17.8 (28/157)
***Leng 2004***	n.a.	7.4	n.a.	n.a.	n.a.
***Lumsden 2000***	47.3	5.7	1.1 (1/95)	n.s.	14.0
***Raju 1994***	57.0	6.0	0.0 (0/40)	n.s.	0.0 (0/40)
***Sculpher 2004***	74.1	5.1	6.2 (18/292)*	27.1 (79/292)**	n.s.
***Summit 1998***	146.0	4.1	32.3 (10/31)	n.s.	32.3 (10/31)‡
**Weighted mean RCT**	**76.9**	**5.1**	**16.1**	**27.1**	**24.6**
**Weighted mean**	**101.3**	**4.8**	**15.9**	**23.0**	**21.7**

n.s.  =  not specified; n.a.  =  not available; * =  percentage of patients with complications; ** =  data derived from original article; ‡ =  number of complications reported.

#### Statistical analysis

All the analyses were done for all included studies together and a sub-analysis is done for the RCTs only. Total costs were calculated for each treatment arm in each study separately. Mean estimated costs with the range (minimum-maximum) were presented. To compare cost and effect pairs between studies, both costs and results were re-calculated to the level of 100 patients per treatment arm. Subsequently, point estimates of cost and effect pairs were plotted in a cost-effectiveness plane. These analyses were performed in Microsoft Office Excel (2003).

## Results

### Characteristics of selected studies

Of all included trials, 6 of the 12 controlled trials concerned randomized controlled trials [11], [12], [17], [20]–[22]. Five studies scored ≥10 points (out of 19) for methodological quality. In the majority of studies (7 out of 12) the costs were analyzed with a cost consequence analysis, mostly from a hospital perspective. No influence of funding or sponsoring was reported. In only two studies, (also) women with a malignant indication for a hysterectomy were recruited. The interventions performed in the trials varied from a simple hysterectomy to a more extended hysterectomy with or without lymphadenectomy. Furthermore, the extent to which the hysterectomy was completed laparoscopically differed from a LAVH to a LSH or a TLH. In total, 2902 patients were included, of which 662 patients underwent a vaginal hysterectomy or were allocated to a vaginal arm. Analysis was performed on 2226 patients; 1213 (54.5%) in the LH group and 1013 (45.5%) in the AH group.

The total direct costs of the LH group ($63,997) were 6.1% higher than the AH group ($60,114). Three studies reported lower costs for the LH group compared with the AH group [15], [18], [20]. The total indirect costs of the LH group ($1,609) were half of the total indirect costs in the AH group ($3,139). A direct relation between costs and included cost components could not be observed.

### Cost components: procedure and hospital stay

From table 4 can be concluded that the mean duration of a laparoscopic procedure ranges from 81.9 minutes [12] to 200.6 minutes [15]. The weighted mean duration of a laparoscopic procedure was 127.7 minutes. In two studies other procedures were performed in addition to the hysterectomy, equally distributed over both arms [18], [19]. Conversely, the shortest mean duration of an AH was 47.3 minutes [12] and the longest mean duration 146.0 minutes [22] (table 4). The weighted mean duration of an AH was 101.3 minutes. The shortest mean hospital stay was 1.5 days after a LH and 2.5 days after an AH. The longest mean hospital stay was 5.1 days and 7.4 days after a LH or an AH, respectively. The weighted mean hospital stay after a LH was 3.6 days (table 4), whereas the weighted mean hospital stay after an AH was 4.8 days (table 5). Overall, in each study the mean hospital stay was lower in the LH group compared to the AH group.

### Effect components: overall and major rate

Overall, the mean complication rate was 15.3% in the LH group and 21.7% in the AH group, with a complication rate difference of 6.4% (table 4 and 5). The mean major complication rate was lower in the LH group (14.3%) than in the AH group (15.9%), with a complication rate difference of 1.6%. An even stronger trend can be seen from the comparison of minor complication, 11.0% in the LH group and 23.0% in the AH, respectively. For the sub-analysis of only RCTs the mean complication rate was 19.9% in the LH group and 24.6% in the AH group, with a complication rate difference of 4.7%. The mean major complication rate was even higher in the LH group (17.8%) compared to the AH group (16.1%), with a complication difference of −1.7% (table 4 and 5).

### Incremental costs and effects

**Figure 2:**
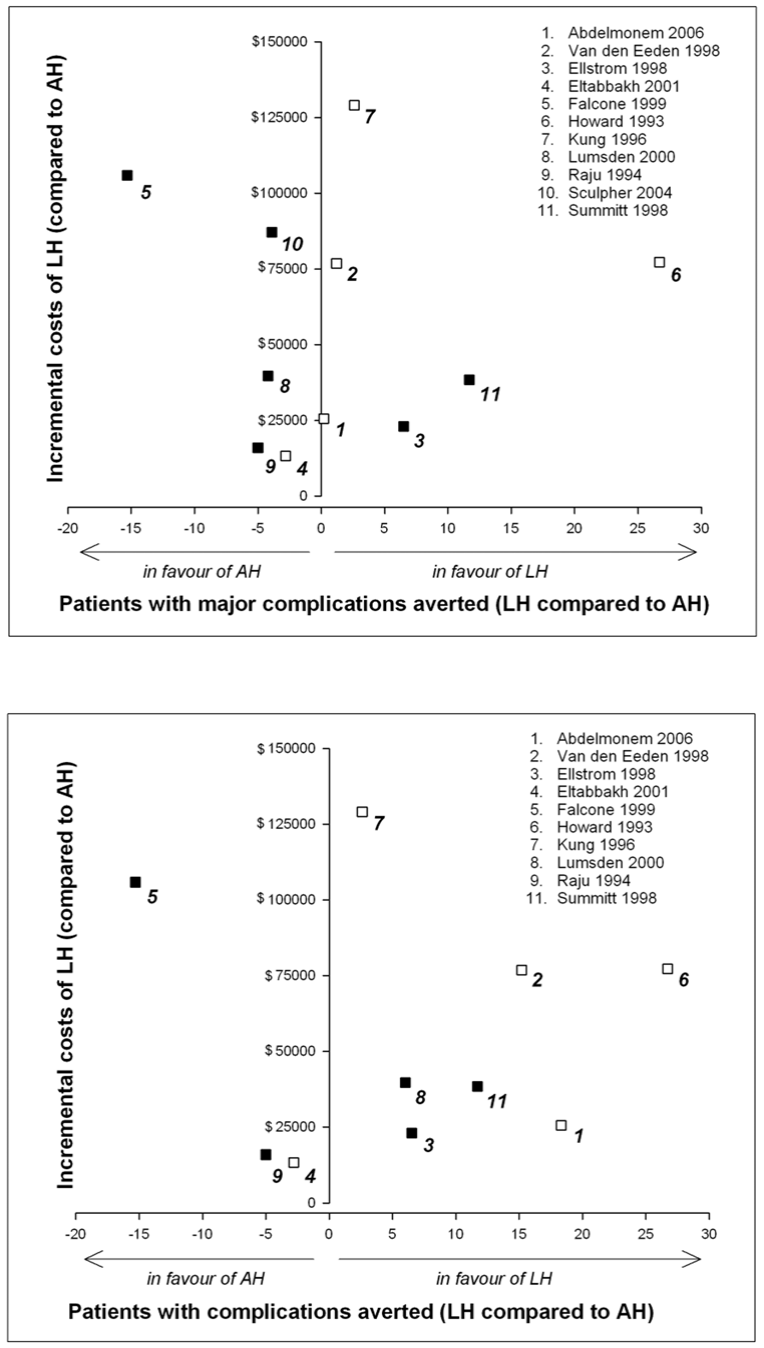
Incremental costs and effects for patients with major complications (A) and all complications (B) per 100 patients ▪ * =  RCT □  =  CCT.*

The estimated mean costs for the procedure were higher in the LH group $2,226 ($1,807–$2,531) than in the AH group $648 ($270–$909). In contrast, mean costs for hospital stay were lower in the LH group ($1,658: $856–$2,795) compared to the AH group ($2,664: $1,426–$3,424). In sum, the total costs in the LH group were $3,884 ($3,130–$4,928) and in the AH group $3,312 ($2,208–$3,767), with a cost difference of $572 between groups. The incremental costs for reducing one patient with complication(s) in the LH group compared to the AH group were $8,938, ($572×100/6.4) re-calculated to a level of 100 patients. When subdivided for major complications, the incremental costs were $35,750 ($572×100/1.6) for reducing one patient with major complication(s). For the sub-analysis of only RCTs, the total costs in the LH group were $3,794 ($3,263–$4,451) and in the AH group $3,277 ($2,208–$3,767), with a cost difference of $517. The incremental costs for reducing one patient with complication(s) in the LH group compared to the abdominal group were $11,000 ($517×100/4.7). Regarding major complications, effects were lower with higher costs in the LH group. Three point estimates are located in the north-east quadrant of figure 2a, indicating that these studies generated extra effects of LH for relatively low additional costs, within a cost ratio range of $2,895 to $3,552 [11], [18], [22]. With regard to overall complications, six studies are located in the north-east quadrant [11], [12], [14], [15], [18], [22] and therefore generating extra effects of LH within a cost ratio range of $1,401 to $6,618 (figure 2b).

## Discussion

For the first time, costs and short term effects were reviewed and calculated between laparoscopic hysterectomy and abdominal hysterectomy in a large group of patients. Cost evaluations for other diseases comparing laparoscopic and open surgery already published, demonstrated that laparoscopic appendectomy, laparoscopic colectomy and laparoscopic cholecystectomy results in decreased hospital stay, lower hospital cost and faster return to work or daily activities than open procedures [29]. Our review showed a similar trend with regard to hospital stay and hospital costs. Twelve trials concerning 2226 patients in total were reviewed, of which five trials scored ≥10 points (out of 19) for methodological quality. As reported by the authors, the direct costs were higher and indirect costs were lower for LH compared to AH. Procedure costs were estimated higher and costs of hospital stay were estimated lower in favor of LH. In the LH group the estimated overall and major complication rate were both lower compared to the AH group, 6.4% and 1.6% respectively. Overall, the incremental cost for reducing one patient with complication(s) in the LH group compared to the AH group was $8,938 and $35,750 for reducing one patient with major complication(s).

Based on this data, it is not clear which costs are really associated with the actual cost difference between both treatment modalities. There is significant variability between studies in the range of cost components covered. Although some authors give reasonable detail on economic data, [11], [12], [15], [21] many give scanty information or do not even specify the method of measuring costs [14], [16]–[20], [22]. Strikingly, total costs were far lower for the Chinese and Taiwanese study [19], [26] compared to the other included studies. Presumably, costs for hospital stay and/or out patient department visits were much lower in Asia than in Western countries. The direct costs were higher for LH, whereas the indirect costs were lower than an AH. Nevertheless, only one study collected data on indirect costs. Importantly, previous reports stated that there might be societal benefits associated with lower indirect cost for LH [7], [16], [22], [30]–[34]. In this analysis, cost drivers considered were the procedure (including use of disposables) and hospital stay. Noticeably, there is a wide range in these main cost drivers between the studies. The wide range is caused by the duration of operation time and the experience of the surgeons. Operating time is related to the disease process (e.g. benign or malignant) and the occurrence of additional procedures. A more extended hysterectomy in case of a malignancy might have influence on the length of hospital stay, as well. Inevitably, some of the studies have compared inexperienced laparoscopic surgeons with experienced ‘open’ surgeons. As surgeons become more experienced with laparoscopic procedures, the length of operating time decreases [35]–[37]. By training of surgeons in laparoscopy and using re-usables instead of expensive disposables, procedure costs for LH can be drastically reduced.

The same diversity as is found in costs between studies can be seen in complication rates. In two studies [15], [22] an overestimation of complication rate might be shown in table 4, since only number of complications were reported and not number of patients with complications. Some studies reported major and minor complications separately [12], [14], [15], [21], with high minor complication rates. A possible explanation for this phenomenon can be the definition of minor or major complications. Standardized criteria for defining minor and major complications should be used in order to adequately compare complication rate [38]. In most of the studies peroperative complications and short-term post operative complications were reported and not long-term complications. We therefore could not specify the occurrence of long-term complications. Besides, the studies were selected on bases of including costs in their report, not on morbidity as effect measure. Since a part of the selected studies were of low methodological quality (<10 points), the reported morbidity rates might dissent from well-designed studies, powered on morbidity. In comparison, in a large randomized controlled trial Garry et al. demonstrated a major complication rate of 9.6% for LH and 6.2% for AH, excluding conversions regarded as complications [39]. Reasons to convert a procedure are to avoid a complication to occur or because of a complication that occurred. In the latter, the complication is already recorded separately. Calculating a mean effect, while some studies showed an opposite effect might give a distorted picture. Again, reasons for this diversity are the experience of the surgeons and the indication of performing a hysterectomy. Inexperience with this advanced laparoscopic procedure results in a higher peri-operative complication rate during the learning curve [7].

The sub analyses of only RCTs indicated that the amount of costs were higher or did not show additional effects when looking at major complication rate. The latter means that implementation of the laparoscopic technique is not cost effective as a standard procedure. Minimization of selection bias and information bias might be explanations for the higher morbidity rates in the LH group and as a result not being cost effective as was found in RCTs compared to all controlled trials. Randomization is important as it helps reduce the possibility of bias [40]. Without randomization, there may be a tendency for researchers to select these participants for particular intervention groups they favor (i.e. selection bias). In this way, systematically different estimates of treatment effects can be yielded [41]. Moreover, most of the RCTs were rigorously set up and monitored to ensure accurate registration of complications and therefore safeguarding a systematic bias in reporting frequency (i.e. information bias) by approach.

However, based on other criteria such as quality of life and survival, LH might be more advantageous than AH. Results of two large RCTs i.e.the GOG Lap2 study [42] and the LACE trial [43] are expected in the near future with quality of life and survival as primary endpoints. This will make more definitive conclusions about these specific outcomes possible. Since the aim of the present review was to globally explore differences in costs and effects between both treatment arms independent of the indication, the analyses of all controlled trials were more important and decisive. Furthermore, because the relative differences were analyzed instead of absolute differences the indication for removal of the uterus and consequently the extent of the procedure was therefore considered less relevant.

On average, an amount of $8,938 has to be invested to reduce one complication when performing a LH in stead of an AH. The explicit quantification of acceptable costs for a given benefit is difficult to define. In other words, what costs are acceptable for reducing one complication? From previous cost reports, a cut of value of $100,000 for reducing one death seems cost effective and therefore acceptable [44]. No reports are available, defining a cut off value for reduction one complication. The National Institute for Health and Clinical Excellence (NICE) has adopted a cost effectiveness threshold range of $40,000–$60.000 per Quality Adjusted Life Year (QALY) [45]. According to this guideline a conservative range of $0–$20,000 to prevent one additional complication represents an acceptable cost effectiveness ratio, considering the (unknown) variation of complications and the consequences of a complication in terms of prolonged hospital stay, re-intervention and patient burden.

### Conclusion

The perception that laparoscopic procedures are more costly than open procedures has been a major reason for the slow acceptance of laparoscopic surgery. Important is whether LH is more effective and more costly, with the added benefit worth the added costs. From this study it can be concluded that the benefits of shorter hospital stay in the LH group might compensate for the increased procedure costs. Laparoscopic hysterectomy points in the direction of cost effectiveness, however a broader perspective is needed, including indirect costs and long term effects as survival and quality of life for determining implications for practice. Currently, a cost effectiveness evaluation of the two surgical approaches is being conducted alongside a large multi-centre RCT [46], complying with all guidelines.

## Supporting Information

Appendix S1 QUORUM checklist
(0.03 MB PDF)

Appendix S2 Characteristics of included studies
(0.15 MB DOC)
