# Supplementary material for: Costs and Effects of Abdominal versus Laparoscopic Hysterectomy: Systematic Review of Controlled Trials
Source: PLoS One. 2009 Oct 5;4(10):e7340. doi: 10.1371/journal.pone.0007340 (PMC2752190; doi:10.1371/journal.pone.0007340)
Supplement: Appendix S2 — Characteristics of included studies (0.15 MB DOC) [file pone.0007340.s002.doc]

**Appendix A: Characteristics of included studies**

| **Study** | **Abdelmonem 2006** |
| --- | --- |
| Aim | Comparison of short term outcome between TAH ,TVH and LH. |
| Methods | Patients were not randomized to surgical approach. Allocation to TAH, TVH or LH was determined based on indication for hysterectomy, uterine size, vaginal access, presence of an adnexal mass, patient preference and surgeon preference. Parallel group design with no blinding. The study included 177 patients. No description of withdrawals or dropouts reported. No intention-to-treat analysis was used. Follow-up: patients were asked to report Physical, psychological and sexual satisfaction rates, evaluated by phone questionnaires at a postoperative time period ranging from 1 to 3 months. A cost consequence analysis was performed from a hospital perspective. Total costs was obtained by review of the hospital billing record after discharge. |
| Participants | 177 women with mean age of 40.3 years, mean BMI of 30 kg/m2 and mean uterine size of 8 weeks, recruited from the University of Louisville Hospital and Norton Hospital in Louisville, Kentucky. Inclusion criteria: benign gynecologic condition and uterine size up to 15 weeks. Exclusion criteria: suspected gynecologic malignancies, uterine size >15 weeks and concomitant surgery, including reconstructive pelvic surgery and stress incontinence surgery. |
| Interventions | AH versus LH [LAVH, LSH, TLH] versus VH  AH arm (n=50), LH arm (n=51), VH arm (n=76). Fourth- or third-year residents under faculty surpervision performed all the surgery. |
| Outcome | Operative time, estimated blood loss, postoperative pain, recovery milestones, complication rates, hospital stay, hospital costs and patient satisfaction |
| Notes |  |
| Quality assessment score | 5 |

| **Study** | **Van den Eeden 1998** |
| --- | --- |
| Aim | Comparison of Qol, utilization and costs for women undergoing elective abdominal, laparoscopically assistd vaginal, or vaginal hysterectomy within a managed-care organization. |
| Methods | Patients were not randomized to surgical approach . Parallel group design with no blinding. All women who underwent an elective hysterectomy (AH, LAVH or VH) for any indication in one of the 17 regional facilities joining the Kaiser Permanente Medical Care Program were asked for participation. Of the large sample of abdominal hysterectomy, 30% patients were randomly selected to participate. Of the 324 women who were initially contacted for this study; 287 (89%) completed the study. The primary reasons for incomplete data were loss to follow-up and participant withdrawal. No intention-to-treat analysis was used.  Follow-up: women were interviewed at baseline regarding quality of life measures and reinterviewed by telephone at day 7, day 14 and day 28 after surgery. Rehospitalization, outpatients visits, rehabilitation or physical therapy visits were obtained until 60 days after surgery. A cost consequence analyses was performed from a hospital perspective. Cost data for utilization were obtained from the cost management information system. |
| Participants | 287 women with a mean age of 47.6 years, mean BMI 28.7 kg/m2, recruited in one of the 17 regional facilities belonging to the Kaiser Permanente Medical Care Program, United States. The final number of study participants represents nearly 10% of the total number of elective hysterectomies performed at these facilities during the study period. Inclusion criteria: women undergoing an elective (i.e. scheduled) abdominal, vaginal or laparoscopically assisted hysterectomy. Exclusion criteria not reported. |
| Interventions | AH versus LH [LAVH] versus VH.  AH (n=164). LH (n=56). VH (n=67). Level of surgeons was not stated. A prospensity score analysis was used to control for all presurgical variables that exhibited significant differences between group. This prospencity score is included as a covariate to implicitly adjust the estimated differences between surgical approaches. |
| Outcome | Quality of life, activity levels, health care utilization, operating time, length of hospital stay, pre,- and postoperative gynecological visits, estimated blood loss, hospital costs |
| Notes |  |
| Quality assessment score | 9 |

| **Study** | **Ellstrom 1998** |
| --- | --- |
| Aim | Comparison of economic consequences and postoperative health status following abdominal and laparoscopic hysterectomy. |
| Methods | Randomization 1:1 ratio by opening one of 150 opaque envelopes. Parallel group design with no blinding. Number of women randomized=143. No dropouts reported. Follow-up: self-assessed questionnaires in a subgroup of patients 1, 3 and 12 weeks after surgery. Economic analysis assessed in a period of 12 weeks. Patients in which the procedure was converted to laparotomy remained in the LH group for follow-up, according to the intention to treat principle. A cost consequence analysis was performed from a societal perspective. |
| Participants | 143 women with a mean age of 48.4 years and a mean BMI of 24.5 kg/m2, recruited from Sahlgrenska University Hospital Sweden. Inclusion criteria: scheduled for abdominal hysterectomy for benign disorders, with a maximum uterus width of less than 11 cm as measured by transvaginal ultrasound and not considered suitable for vaginal hysterectomy. Exclusion criteria not reported. |
| Interventions | AH versus LH [LAVH].  AH (n=72). Patients randomized to laparotomy were operated on by two out of ten surgeons of senior registrar grade attached to the department and well trained in abdominal hysterectomy. LAVH (n=71). The patients randomized to LH were operated on by tow out of the five surgeons of senior registrar grade attached to the department and specially trained in the laparoscopic procedure. |
| Outcome | Postoperative health status, quality of life, operating time, hospital stay, sick leave, outpatients visits, direct costs (hospital costs) and indirect costs (loss of production value). |
| Notes |  |
| Quality assessment score | 13 |

| **Study** | **Eltabbakh 2001** |
| --- | --- |
| Aim | Comparison of surgical outcome, cost and quality of life among patients treated with laparoscopy or laparotomy. |
| Methods | No randomization, but controlling by identifying all surviving women with clinical stage I endometrial carcinoma or uterine sarcoma who underwent TAH and BSO at the division of Gynecologic Oncology, University of Vermont in the 2 years before the study period and who continued to be followed. Follow up: telephone interview by a nurse with a median duration of 17 months among LAVH group and 40 months among the TAH group. No drop outs reported. In case of conversion, patients were kept in the laparoscopy for further analysis, according to the intention to-treat principle.  A cost consequence analysis was performed from a hospital perspective. The cost of the procedures was based on the amount billed and not the amount actually received. The cost was adjusted according to date and any additional procedures performed. |
| Participants | 147 women with a mean age of 60.9 years and mean BMI of 30.1 kg/m2, recruited from the division of gynecologic oncology at the University of Vermont. Inclusion criteria: women with clinical stage I endometrial carcinoma or uterine sarcoma, who could tolerate laparoscopic surgery. Exclusion criteria: patients who refused laparoscopy or had one or more of the following conditions were excluded from the study; 1). macroscopic cervical involvement by tumor, 2) uterus >12 weeks unless enlarged by subserous myomas, 3) severe cardiopulmonary disease precluding use of the Trendelenburg position, 4) severe hip disease precluding use of the dorsal lithotomy position, 5) BMI >60.0 kg/m2, 6) prior pelvic or abdominal radiation. |
| Interventions | AH versus LH [LAVH] with BSO and peritoneal washings +/- lymphadenectomy. (partial omentectomy was performed in women with papillary serous histology). AH (n=57). LAVH (n=90). The senior investigator performed surgery on all patients with a chief resident as the first assistant. In the majority of patients, the chief resident performed the vaginal portion of the procedure with the senior investigator as the first assistant. |
| Outcome | Operating time; estimated blood loss; length of hospital stay; pain medication; per,- and postoperative complications; costs |
| Notes |  |
| Quality assessment score | 10 |

| **Study** | **Falcone 1999** |
| --- | --- |
| Aim | Comparison of operative time, length of hospital stay, postoperative recovery, return to work and costs for women undergoing laparoscopically assisted vaginal hysterectomy or abdominal hysterectomy. |
| Methods | Randomisation: assigned according to a computer-generated randomization schedule with random block sizes. Parallel group design with no blinding. Number of women randomized: 48, number analysed: 44. Four women withdrew before surgery (3 AH group and 1 LH(a) group). Analysis was by intention to treat. Follow-up: dialy diary for 6 weeks. A cost minimization analysis was performed from a hospital perspective. The hospital costs of the procedures were assessed through the hospital accounting system. |
| Participants | 48 women with a mean age of 43.3 years and a mean BMI of 29.3 kg/m2. Participants were recruited from Cleveland Clinic Foundation, Ohio USA. Inclusion criteria: scheduled for abdominal hysterectomy for benign disease. Exclusion criteria: pelvic mass size greater than 2 cm below the umbilicus; concomitant incontinence or pelvic reconstructive procedures required. |
| Interventions | AH versus LH [LAVH]  LAVH arm (n=24): performed by senior author with assistance from pelvic surgery fellow or resident. AH arm (n=24) |
| Outcome | Operative time; blood loss; length of hospital stay; intraoperative complications; postoperative pain; return to work/normal activities, costs. |
| Notes |  |
| Quality assessment score | 11 |

| **Study** | **Howard 1993** |
| --- | --- |
| Aim | Comparison between laparoscopically assisted vaginal hysterectomy with total abdominal hysterectomy |
| Methods | No randomization, but assignment to either LAVH or TAH group based on the attending physician scheduled for the case. No intention to treat analysis was used. Follow up not reported.  A cost consequence analysis was performed from a hospital perspective. Hospital costs were obtained from the accounting office. |
| Participants | 30 women, of which mean age, weight and height were not different in the two groups (not further specified). Participants were recruited from the Rochester General Hospital, New York, USA.  Inclusion criteria: enrollment in the study only if there was a relative contra-indication to vaginal hysterectomy based on a preoperative diagnosis of endometriosis, chronic pelvic pain, adnexal disease, adhesive disease, prior abdominopelvic surgery or enlarged uterine size (12-18 weeks size). Exclusion criteria not reported. |
| Interventions | AH versus LH [LAVH]  AH arm (n=15); TAHs were performed on patients scheduled under the supervision of five other faculty members. LAVH arm (n=15); LAVHs were performed on all patients scheduled under the supervision of the authors. At time of hysterectomy, 36 (LAVH; n=18 and TAH; n=18) additional operative procedures were performed. |
| Outcome | Postoperative pain medication use, operating time, length of hospital stay, estimated blood loss, complication rate, VAS, hospital costs. |
| Notes |  |
| Quality assessment score | 2 |

| **Study** | **Kung 1996** |
| --- | --- |
| Aim | To compare the differences among intraoperation-related parameters, postoperative convalescent parameters and hospital costs between laparoscopically assisted vaginal hysterectomy and total abdominal hysterectomy. |
| Methods | No randomization, but the decision in favor of hysterectomy, either TAH or LAVH, was made by the on-service attending doctor who was the patient’s primary care provider. No intention-to-treat analysis used, as converted patients (n=6) were excluded for further analysis. No dropouts reported. Follow up not reported. A cost study was performed from both hospital and insurance perspective. |
| Participants | 301 women with a mean age of 44.4 years and a mean BMI of 24.5 kg/m2, recruited from the Chang Gung Memorial Hospital, Taiwan. Inclusion criteria: patients with a diagnosis of benign uterine disease who were scheduled for hysterectomy were selected if they met the following criteria; 1) estimated uterine size less than 16 weeks of gestation; 2) no pre-existing cardiopulmonary dysfunction, poor control of diabetes mellitus or systemic diseases; 3) bimanual pelvic examination to confirm good mobility of an enlarged uterus indicated removal would be difficult via conventional vaginal hysterectomy; 4) no history of prior complex abdominopelvic surgeries excluding appendectomy, tubal sterilization, cesarean section or laparotomy for benign ovarian disease. Exclusion criteria: Subjects were excluded from this study if: 1) associated nongynecologic procedures such as inguinal herniorrhaphy, incidental appendectomy, or breast biopsy for a suspected lesion were required; 2) patients in the LAVH group misunderstood or objected to laparoscopic surgery after counseling; 3) patients in the TAH group requested the laparoscopic approach but their on-service physicians were unfamiliar with LAVH techniques. |
| Interventions | AH versus LH [LAVH]  AH arm (n=157), LAVH arm (n=144). A total of five attending doctors, committed to recruiting patients for purposes of comparison between the two procedures, performed all the hysterectomies in the study. The percentage of concomitant adnexectomy was significantly lower with the LAVH approach. |
| Outcome | Conversion rate, operating time, estimated blood loss, pain medication use, complication rate, length of hospital stay, hospital costs |
| Notes | The cost of disposable staples and instruments is not covered by insurance in Taiwan. To save the patient extra charges and approximate the instrumental requirements of the TAH, only reusable instruments and common electrosurgical instruments for the LAVH were used. |
| Quality assessment score | 6 |

| **Study** | **Leng 2004** |
| --- | --- |
| Aim | To evaluate the effects of total abdominal hysterectomy (TAH), laparoscopic hysterectomy (LH) and transvaginal hysterectomy (TVH) on quality of life, cost and health care uitilization. |
| Methods | Patients were not randomized to surgical approach. Allacation to intervention was not specified. Parallel group design with no blinding. The study included 55 patients. Three dropouts were reported. No intention to treat analysis was performed. Follow-up: WHO quality of life questionnaires completed preoperatively, 4 days, 14 days and 28 days postoperative. A cost analysis from a societal perspective (not stated in methods). Total costs were obtained from hospital billing charts and indirect cost from the self reported questionnaires. |
| Participants | 52 women, of which mean age and BMI were not different in the three groups (not further specified). Participants were recruited from the Peking Union Medical College, Being, China.  Inclusion criteria: married, employed women between 30-55 years without other co-morbidity. Exclusion criteria not reported. |
| Interventions | AH versus LH  AH arm (n=19), LH arm (n=20). Level of surgeons was not stated. |
| Outcome | Quality of life, health care utilization, hospital stay, frequency of outpatient department visits. |
| Notes |  |
| Quality assessment score | 5 |

| **Study** | **Lumsden 2000** |
| --- | --- |
| Aim | To determine the safety, cost effectiveness and effect on quality of life of laparoscopic-assisted vaginal hysterectomy (LAVH) compared with total abdominal hysterectomy (TAH) in the management of benign gynaecological disease. |
| Methods | Randomisation: performed by the research nurse using a computer-generated schedule. Parallel group design with no blinding. Number of women randomized: 200, number analysed: 190. Seven women did not attend for operation and the case records were not available for a further 3 women. Analysis was stated as intention to treat, but not all randomized participants were analysed. Follow-up: diary of recovery ‘’milestones’ 4 weeks after surgery. EQ health questionnaire completed at one, six and twelve months after surgery. A cost consequence analysis was performed from a hospital perspective. A single set of costs was applied to each unit of resource used to provide a NHS cost for each women. |
| Participants | 190 women with a mean age of 41.9 years and a mean BMI of 26.5 kg/m2, recruited from three hospitals in Glasgow, Scotland. Inclusion criteria: scheduled for AH for benign gynaecological disease and they were not suitable for VH because of a uterine size in excess of 14 weeks or a requirement for oophorectomy. Exclusion criteria: those patients in whom hormone replacement therapy was not appropriate. |
| Interventions | AH versus LH [LAVH].  AH arm (n=95) and LAVH arm (n=95). All procedures were performed by 5 consultant gynaecologists who have undertaken a minimum of 50 LH procedures. |
| Outcome | Length of operation; length of hospital stay; readmissions; reinterventions; blood transfusions; complications; pain medication use; quality of life; hospital costs |
| Notes |  |
| Quality assessment score | 17 |

| **Study** | **Raju 1994** |
| --- | --- |
| Aim | Identification of differences in peri-operative outcome of women undergoing hysterectomy with bilateral salpingo-oophorectomy performed either by abdominal hysterectomy and bilateral salpingo-oophorectomy or by laparoscopic-assisted salpingo-assisted salpingo-oophorectomy and vaginal hysterectomy. |
| Methods | Randomization by sealed envelopes containing computer generated block randomization numbers. Block size of 10. Parallel group design with no blinding. Number of women randomized: 80, number analysed: 80. No dropouts reported. Intention-to-treat analysis not stated, but 2 converted patients were kept in laparoscopy arm. Follow-up: patients were questioned about their general state of health and return to normal activities 6 weeks after surgery. A cost minimization analysis was performed on the major points of difference between either operation (disposables, length of hospital stay) from a hospital perspective. |
| Participants | 80 women with mean age 45.7 years and a mean BMI of 25.3 kg/m2, recruited from St. Thomas’ Hospital London, UK. Inclusion criteria: scheduled for hysterectomy and bilateral oophorectomy for benign conditions. Exclusion criteria: morbid obesity, uterus larger than 14 weeks gestation size, or uterovaginal prolapse. |
| Interventions | TAH with BSO versus LH [LASOVH]  LAVH arm (n=40) : All laparoscopic procedures performed by one of the two authors. AH arm (n=40): Operations performed by one of the authors or by another surgeon of senior registrar grade using a standard technique. |
| Outcome | Operating time, blood loss, hospital stay, postoperative analgetics, complications, recovery time |
| Notes | All the cannulae used for laparoscopy were of the reusable type. |
| Quality assessment score | 7 |

| **Study** | **Sculpher 2004** |
| --- | --- |
| Aim | Assess the cost effectiveness of laparoscopic hysterectomy compared with conventional hysterectomy (abdominal or vaginal). |
| Methods | Randomization: 2:1 imbalance randomization method, by telephone and performed with a computer-generated programmed. Allocation to abdominal or vaginal trial by surgeon. Parallel group design with no blinding. Total number of women randomized; 1380 of which 859 patients in abdominal trial. Number analysis: 1346. 34 withdrew before surgery was undertaken. Analysis by intention to treat (ITT) results were confirmed using a per-protocol analysis and the ITT analysis included all randomized patients, even those who did not have an operation. Follow-up: questionnaires (EQ-5D) at 6 weeks, 4 months and 1 year after surgery. A cost utility analysis was performed from a health care provider perspective. Key unit costs were used to value resource use. |
| Participants | 1380 women with a mean age of 41.3 years and mean BMI of 26.4 kg/m2, recruited from 28 centres throughout the UK and 2 centres in South Africa. Inclusion criteria: women who needed hysterectomy for non-malignant conditions. Exclusion criteria: confirmed or suspected malignant disease of any part of the genital tract; 2^nd^ or 3^rd^ degree uterine prolaps; a uterine mass greater than the size of a 12-week pregnancy; any associated medical illness precluding laparoscopic surgery; a requirement for bladder or other pelvic support surgery and patient refusal of consent for the trial. |
| Interventions | Abdominal trial: AH versus LH [LAVH, LSH, TLH]  AH arm (n=573), LH arm (n=286). Surgeons recruited had to have performed at least 25 of each type of procedure. Surgeons of all grades and experience participated. |
| Outcome | Complications, blood loss; pain; analgesia requirement; length of surgery; length of hospital stay, costs |
| Notes |  |
| Quality assessment score | 15 |

| **Study** | **Summit 1998** |
| --- | --- |
| Aim | Comparison of intraoperative and postoperative outcomes between laparoscopically assisted vaginal hysterectomy and abdominal hysterectomy among patients who are not eligible for vaginal hysterectomy. |
| Methods | Randomisation: a computer-generated randomization list, with each surgical assignment placed in consecutive sealed envelopes. Parallel group design with no blinding. Number of women randomized: 65. Number of women analysed: 63. Two other women who were randomized refused their assigned procedure and were removed from the study. Converted patients (n=3) remained in the laparoscopic group for the purpose of data analysis, according to the ITT principle. Follow-up: All patients were seen 2 and 6 weeks postoperatively in the outpatient office. A cost analysis was performed from a hospital perspective. (not stated in methods). |
| Participants | 63 women with mean age 41.1 years, recruited from three participating research centres, USA. Inclusion criteria: indication for hysterectomy and 1) age at least 18 years; 2) a working telephone in the home; 3) available support person in the home for 48 hours after surgery and 4) understanding of the postoperative instructions. Exclusion criteria: medical conditions requiring in-hospital monitoring, known cervical or endometrial cancer, absolute contraindications to operative laparoscopy, including 1) uterine leiomyomas or pelvic masses greater than 18 gestational weeks in size; 2) conditions making them intolerant for anaesthesia; 3) severe bleeding disorders; 4) acute peritonitis of the upper abdomen with severe distention, or 5) a midline abdominal hernia. |
| Interventions | AH versus LH [LAVH]  AH arm (n=31). LAVH arm (n=34). The surgical procedures were performed similarly at all institutions. All operations were performed by gynaecology residents, with attending physicians (investigators) assisting. |
| Outcome | Operating time; length of hospital stay; sick leave; estimated blood loss; complication rate; hospital costs. |
| Notes |  |
| Quality assessment score | 11 |
